# Statistically elucidated responses from low-signal contrast mechanisms in ultrafast electron microscopy

**DOI:** 10.1063/4.0000751

**Published:** 2025-06-12

**Authors:** Spencer A. Reisbick, Alexandre Pofelski, Myung-Geun Han, Chuhang Liu, Eric Montgomery, Chunguang Jing, Kayla Callaway, John Cumings, June W. Lau, Yimei Zhu

**Affiliations:** 1 Condensed Matter Physics and Materials Science Department, Brookhaven National Laboratory, Upton, New York 11973, USA; 2 Department of Physics and Astronomy, Stony Brook University, Stony Brook, New York 11794, USA; 3 Euclid Techlabs LLC, 365 Remington Blvd., Bolingbrook, Illinois 60440, USA; 4 Department of Materials Science and Engineering, University of Maryland, College Park, Maryland 20742, USA; 5 Office of Data and Informatics, National Institute of Standards and Technology, Gaithersburg, Maryland 20899, USA

## Abstract

The emergence of ultrafast electron microscopy (UEM) has enabled the discovery of strongly correlated dynamic mechanisms, including electron–phonon coupling, structural phase transitions, thermal transport, and electromagnetic deflection. Most UEM systems operate stroboscopically, meaning that the technique is susceptible to artifacts, mistakes, and misinterpretation of the data due to extensive experimental effort. In contrast to the ultrafast designation, data acquisition is extraordinarily slow because the electron beam has significantly reduced signal compared to traditional transmission electron microscopy due to pulsing the electron beam. Consequently, the sample may drift, tilt, or undergo irreversible structural changes that are independent of the time-resolved dynamics throughout the experimental time frame. Furthermore, these datasets require significant user interpretation that can be problematic when proper controls are not implemented thoroughly. Here, we demonstrate a new algorithm designed to separate ultrafast structural dynamics from long-term artifacts using a LiNbO_3_ sample experiencing electrically driven surface acoustic wave propagation. Additionally, we provide examples of the impact of user bias when analyzing the data and provide a methodology, which enables the extraction of time-resolved responses when the image signal is extraordinarily low. Overall, the goal of this publication is to provide methods that validate the experimental results and reduce researcher biases during UEM data interpretation.

## I. INTRODUCTION

Ultrafast electron microscopy (UEM) has become a powerful technique used to image materials dynamics with spatiotemporal resolution down to the nanometer-femtosecond regime.^1–6^ The technique was derived by combining the principles of ultrafast spectroscopy and transmission electron microscopy (TEM).^3^ At the most fundamental level, UEM operates by creating ultrashort electron pulses that image the response of a material being perturbed by a synchronized periodic excitation.^1,3,4,7,8^ UEM has evolved from this simple analog to a myriad of techniques, including different probes and pumps, and probing mechanisms.^9–17^ The two most prominent forms are laser-driven and electrically driven UEM, which differ in the pulse electron generation mechanisms. For most laser-driven systems, the electron pulse width is correlated with the laser pulse duration (few fs, assuming no gating or other compression techniques), while electronically generated electron packets depend on the sweeping frequency and velocity of a microwave cavity (few ps).^16,18–20^ The electrically driven variation of UEM still requires improvement in temporal resolution to achieve what is attainable in laser-driven systems but provides a simpler setup when exciting electronic systems.^12,14–17^ Additionally, the larger reason to use either technique is dependent on the physical phenomenon in question (i.e., thermal transport—laser excitation vs device operation—electrical excitation). Nonetheless, the imaging capabilities of laser-driven and laser-free techniques using a pulsed electron beam are comparable, and both exceed the spatial resolutions required for most nanoscale experiments (sample stability under excitation at these spatial resolutions still requires significant advancement).^17,21–24^ For electrically driven systems, dedicated sample holders and instrumentation including RF pulsing cavities and DC beam manipulators have been developed to create pulsed electron beams.^1,6,9,25–31^ The excitation sources have also expanded to include electrical excitations, including RF, AC, and DC in conjunction with traditional laser methods.^16,17,32,33^ The primary benefit of electrical excitation on the sample is the ability to perform *in operando* experiments on devices and the ability to operate at frequencies above a few hundred megahertz, hence shorter acquisition times.^16,17^ In electrically driven systems, the excitation profile is less limiting because it is possible to have a controlled, time-varying potential across the sample.^34,35^ When these excitations are synchronized to a pulsed electron beam, it enables the elucidation of dynamics occurring as fast as a few picoseconds.^12,17,33^ Overall, the development of UEM has pushed the technique to allow numerous methods to explore various materials applications.

Some of the preliminary experiments that allowed UEM to emerge as a promising technique include the metal-insulator transition in VO_2_, acoustic phonon propagation in layered materials, and structural lattice distortions in gold.^1,4,5,25,27,30,36–40^ Since then, the variety of materials has expanded to include materials that require more complicated imaging techniques such as magnetic, ferroelectric, and nanoresonating materials.^2,30,41–50^ However, the largest problem for UEM experimentation is the length of time required to collect the data, making it prone to misinterpretation and susceptible to artifacts. Specifically, UEM experiments require a pulsed electron beam with intensity ranging from 10^−4^ and 10^−9^ of the continuous beam, meaning that matching the contrast of conventional TEM can require a ×10^6^ times longer exposure.^20,51^ Because of the extremely weak signals and uncontrollable experimental instabilities, the ability to generate a high-quality dataset can be extremely difficult. Even when the signal can be acquired in 10–100 s, UEM image sequences often exceed 1000 frames, so it is common for UEM experiments to extend beyond 10 h.^52^ Tuning the number of images and acquisition times can reduce these experiment lengths, but this comes at the cost of low-signal-to-noise or choppy image sequences. Luckily, technological advances, such as direct electron detectors and higher frequency pulser/lasers, have made significant improvements in data acquisition during UEM experiments, drastically reducing some of the obstacles that have been present in the field. Even with these improvements, the competition between image quality and experimentation time is an ever-present problem in the UEM community.

**FIG. 1.**
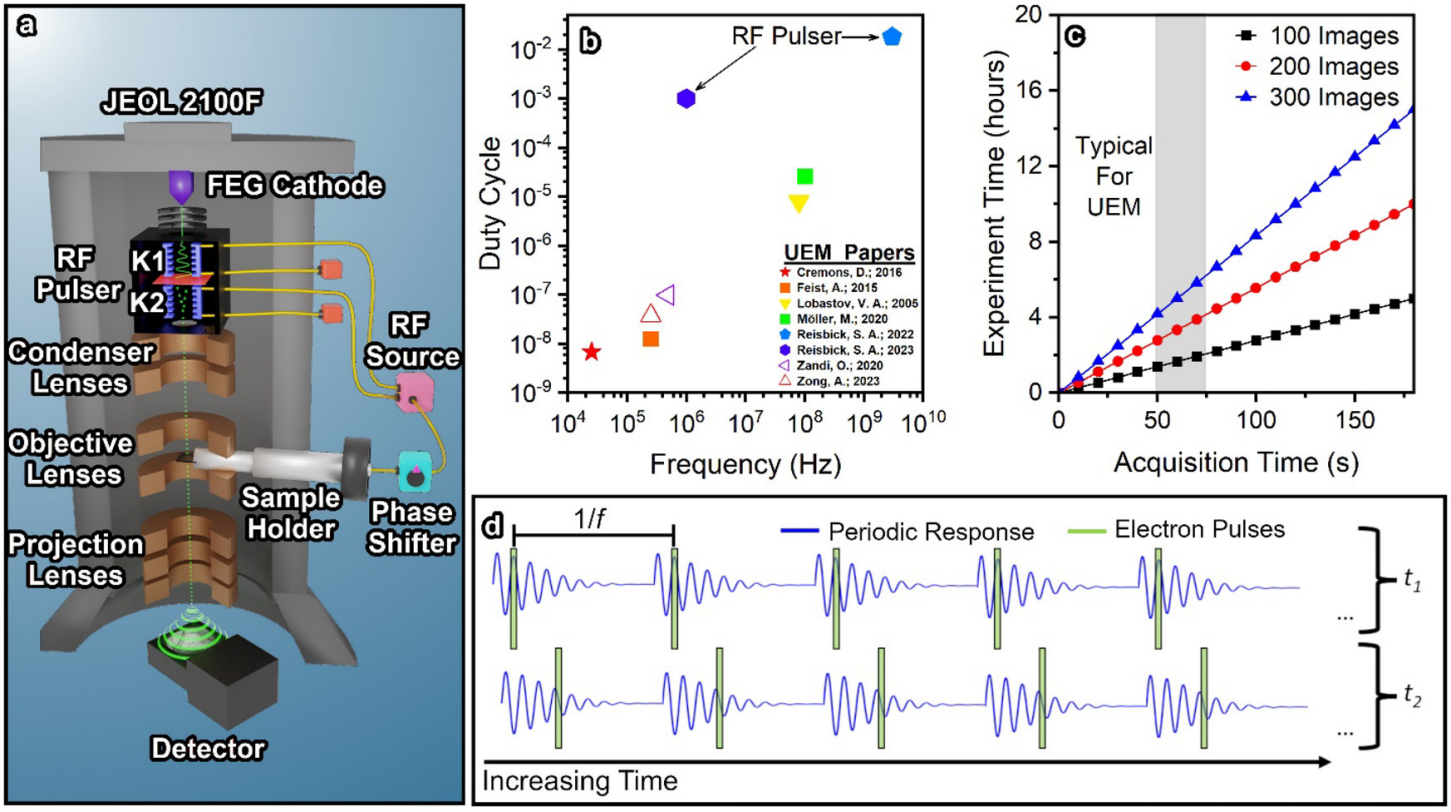
Explanation of long acquisitions and experimentation times during UEM. (a) Schematic of the UEM at Brookhaven National Laboratory constructed by inserting a RF pulsing cavity below the FEG cathode of a traditional JEOL 2100F microscope. The pulsing cavities are controlled by an RF source, which is coupled to the sample excitation and delayed by an electrical phase shifter. (b) Comparison of the frequency and duty cycle for recent UEM experiments from seven different research groups. Note that duty cycle represents the percentage of time that the sample interacts with the electron beam and does not take into account the brightness or other illumination factors. (c) Expected experimentation length for datasets of different frame quantities and acquisition times. For most experiments, the acquisition time is around 60 s, and a minimum of 50–200 images are required. (d) Representation of the imaging electron arriving at the sample during UEM experiments with respect to the induced structural dynamics. The blue line represents a periodic dynamic process, and the green box represents the window of the electron beam as it interacts with the sample. For each acquired image, only the portion of the dynamic process within the window reaches the detector, causing prolonged experiments due to the reduced total signal. At higher frequencies, the impact is reduced because there are more electron pulses (assuming the same pulse duration and brightness).

Here, we discuss a novel processing algorithm coupled to an acquisition technique that separates artifacts in the UEM data from the time-resolved results, specifically, when the images contain low signal. Ironically, most of the flaws with data collection arise from the fundamental principles essential to the UEM workflow. The two most important aspects of a successful UEM experiment are having a pulsed electron beam (probe) that is perfectly synchronized to a periodic sample excitation (pump) while capable of controlling the variable delay between them.^3,4^ Consequently, the pulsed probe throws away over 99.99% of the imaging signal requiring extremely long acquisitions.^53,54^ Second, image sequences require many frames to capture the *in situ* dynamics sufficiently, causing very long experiment lengths.^55^ Figure 1 contains a schematic of the UEM instrumentation at Brookhaven National Laboratory (instrumentation has been described elsewhere) where an RF pulser is our method for pulsing the electron beam.^12,14–17^ Additionally, Fig. 1 contains the duty cycles used in a sampling of recent UEM experiments and the total time required for a typical measurement.^2,3,16,17,31,33,42,56^ Note that here, the duty cycle represents the percentage of time that electrons are interacting with the sample and does not take the brightness of the beam into account. The long experimentation time manifests UEM problems beyond researcher fatigue.^57^ In between image acquisitions, the sample/electron beam can drift, lenses can fluctuate, or power surges can cause image sequence discontinuities. These problems can be present in single images or emerge gradually through an image sequence acquisition, depending on the event. For example, sample drift of one pixel per 5 min would be undetectable until post-processing, whereas an instantaneous arc in the electron gun would cause a dramatic intensity change between two images. In both cases, the experimental data contain obvious flaws, but through the developments in this publication, we demonstrate methods and analysis procedures that enable the extraction of useful time-resolved dynamics from these noisy artifacts in low-signal datasets. We also push to reduce the amount of researcher bias inherent to UEM interpretation through a series of analytics.

The largest consequence of time-resolved experiments is that substantial portions of the electron beam are discarded due to the pulsing process. Electrically driven UEM is capable of higher frequencies (up to 12 GHz) at the cost of longer pulse durations. In this article, we expand on a frequently used control technique that should be routinely performed across the ultrafast field that randomizes the data acquisition to eliminate artifacts. Then, we implement a novel algorithm that normalizes UEM data and extracts convoluted dynamics from chaotic and low-signal measurements during post-processing by developing a metric that separates the time-resolved material responses from instrumentation induced artifacts. Additionally, we discuss researcher bias in UEM experiments and provide one simple example of data interpretation enabled by the aforementioned metric.

## II. METHODS

The Brookhaven National Laboratory UEM is a 200 kV JEOL JEM-2100F^^*^^ modified with a Euclid Techlabs UltraFast Pulser^*^ (hereafter referred to as the pulser) installed directly below the emission chamber.^16,17^ Images were acquired using a Dectris QUADRO^*^ with a custom software dedicated to repetitive acquisitions. One channel of a high voltage dual channel pulse generator (Berkeley Nucleonics^*^) produced a 100 MHz signal oscillating between +40 and 0 V at K1 [see Fig. 1(a)] to pulse the electron beam. The second channel was connected to a custom RF-compatible TEM holder with transmission efficiency around 80% up to 12 GHz.^17^ The excitation was a 5 V (oscillating from −2.5 to 2.5 V) AC signal at a 200 MHz frequency (double the frequency of the probe to account for forward and backward sweeping across the chopping aperture). The sample was a LiNbO_3_ wedge lamella that was a few micrometers thick with a section near the sample edge thinned to 100 nm. An interdigitated transducer (IDT) was patterned directly onto a bulk portion of the LiNbO_3_ sample so that when excited with the 200 MHz excitation, surface acoustic waves (SAWs) were induced that traveled toward the thinned region. Images acquired during long-term stabilization measurements used 60 s exposure times, while time-resolved experiments were achieved at 15 s. The image sequence of the LiNBO_3_ response required approximately 1.5 h to collect 91 images spanning 5 ns (0–2*π* phase for 200 MHz) with 27 ps (2°) steps. Time-resolved experiments were acquired using a 40 *μ*m objective aperture to emphasize bend contour contrast. All processing was completed using the signal processing toolboxes available in MATLAB and a custom graphical user interface that executed the procedures described within.

Certain commercial equipment, instruments, or materials are identified in this presentation to specify the experimental procedure adequately. Such identification is neither intended to imply recommendation or endorsement by the National Institute of Standards and Technology nor intended to imply that the materials or equipment identified are necessarily the best available for the purpose.

## III. RESULTS AND DISCUSSION

**FIG. 2.**
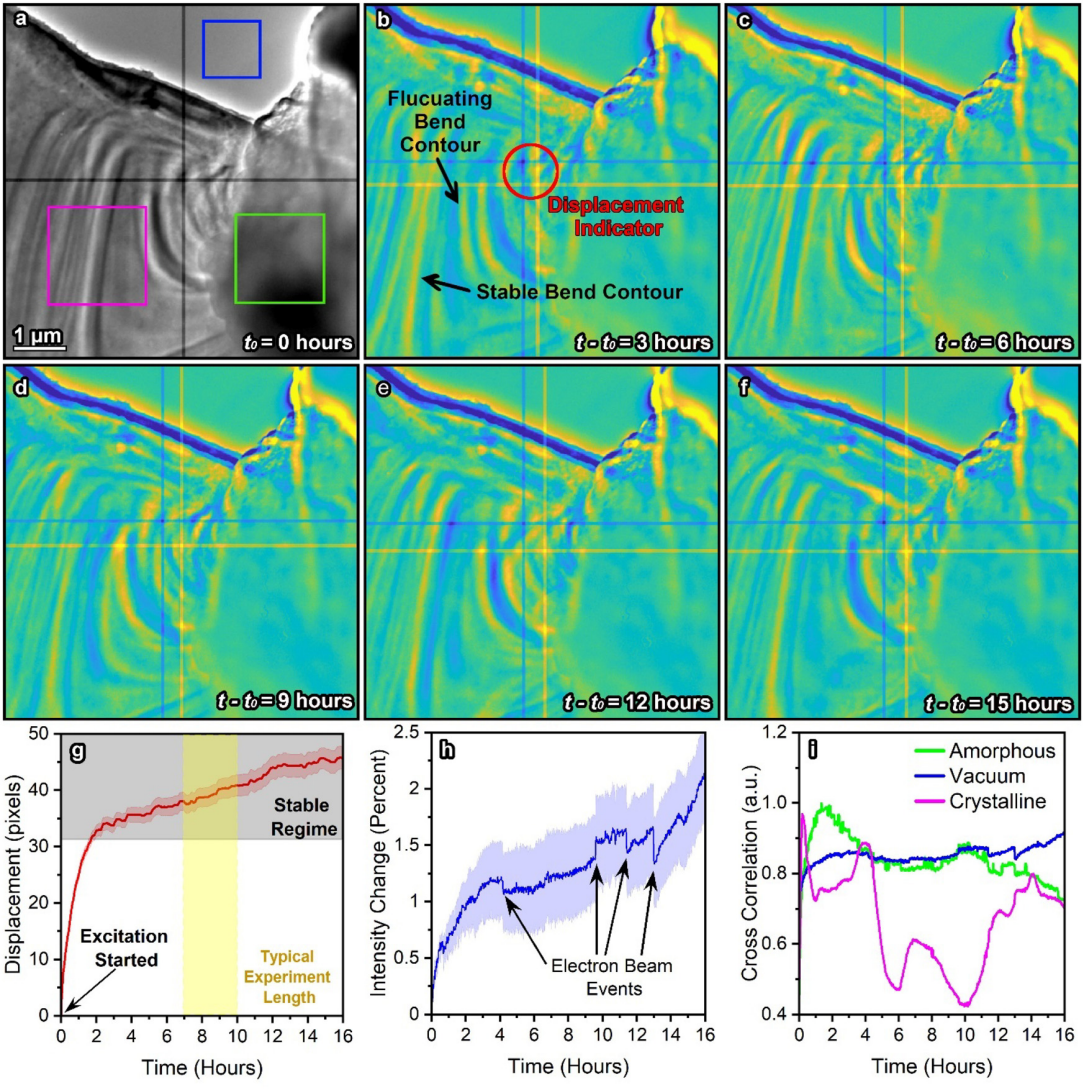
Long-term sample and beam motion of a LiNbO_3_ crystal under standard UEM operating conditions without changing the delay. (a) Reference image of the LiNbO_3_ crystal at the start of the experiment when the pulsed electron beam and 200 MHz AC excitation were activated. The crosshair in the center of the image is a by-product of the detector and intentionally not corrected so that its position could be used during template matching to track the displacement. (b)–(f) Difference images subtracting the designated time point from the reference image (t_0_) where blue and yellow represent intensity decrease and increase, respectively. Each difference image contains two crosses from the reference image (yellow) and the time-resolved image (blue). Note that two bend contours, one that remains stable and one that fluctuates, are indicated for visualization purposes. The entire image sequence can be viewed in supplementary material Video 1.^62^ (g) Displacement of the sample over time where the yellow and gray regions represent the timeframe for a typical experiment and where the sample stabilized, respectively. (h) Change in the average intensity of the electron beam over time taken from the blue square in (a). Note that there is a steady increase in the intensity of the electron beam and numerous beam events (i.e., cathode instabilities or minor sparking in the gun) that cause sharp changes in illumination. (i) Cross correlation taken from crystalline, amorphous, and vacuum regions of the image sequence designated by the color-coded ROIs in (a). Note that the sample contrast motion observed in the crystalline region is all known to be artifacts caused by the longevity of UEM experimentation because the delay between the pump and probe was not adjusted during data collection. Therefore, all of the measured phenomena in these plots are artifacts, which have the potential to convolute the time-resolved dynamics present in the materials. The error ranges in (g) and (h) are the standard deviation of three repeat measurements in different areas.

When UEM experiments are being conducted, the researcher must be cognizant of the long-term instabilities in the microscope that modifies imaging conditions or the macrostructure of the sample, while each image is acquired. Due to the length of these experiments, it is easy to misconstrue artifacts that are consequences of beam induced sample changes or adjustments in beam conditions that can be misperceived as time-resolved dynamics. Figure 2 contains a LiNbO_3_ lamella being excited under typical UEM operation for over 16 h, except that each image is acquired at the same delay position (full video in supplementary material Video 1).^62^ Obviously, there should be no differences in the acquired images when the delay between the pump and probe is not adjusted. However, supplementary material Video 1^62^ and Fig. 2 demonstrate enormous changes in the sample due to the combination of lengthy acquisition times, electron beam damage, and constant electrical excitation. Specifically, the lamella was positioned so a vacuum region, crystalline region, and amorphous contaminant were all visible in the field of view. Figures 2(b)–2(f) contain difference images comparing snapshots at different hours after the reference image [Fig. 2(a)]. The cross in the grayscale image is a by-product of the four on-camera chips combined to form the full detector, usually removed during post-processing. Here, we quantify the displacement of the sample by measuring the shift of the cross when the vacuum-crystal interface is template matched to be stationary. Figure 2(g) demonstrates the 16 h sample displacement starting when the electrical excitation was initiated on the LiNbO_3_ lamella. Note that this sample motion is a random process and is not repeatable, and, thus, it is necessary to deconvolute it from time-resolved, excitation driven results. Figures 2(h) and 2(i) contain cross correlation measurements of various regions of the sample during the experimentation time as described elsewhere.^58^

The control experiment in Fig. 2 portrays the excellent stability of our UEM system shown by less than 2% fluctuation in the electron beam emission over 16 h and a sample drift rate of 16 nm per hour in the stable regime (416 nm per hour in the first hour). The excitation on the sample also creates a more extreme environment that amplifies the motion. Notably, when the electron beam became pulsed, there was a 1% rise in intensity in the first 4 h. Most UEM experiments require waiting for the sample to equilibrate so that drift and nonreversible changes to the system are minimized; for us, this time is usually 1–2 h. Once stability is achieved, there is minimal sample drift and beam fluctuation for about 6 h. Unfortunately, beam-related events are unpredictable [shown by spikes in Fig. 2(h)], so determining the optimal time for an experiment still requires chance. Aside from illumination changes and sample motion, the crystalline region underwent 3–4 different tilt orientations during the experiment. Because the goniometer was not intentionally translated, the tilting was likely due to thermalization or holder instabilities. Consequently, these must be artifacts because the phase delay was held constant. Therefore, the experimentation length and stability requirements of UEM experiments can make analysis and data collection very difficult to navigate. Next, we present a data collection methodology and analysis procedure that enable the extraction of low-signal convoluted dynamics.

**FIG. 3.**
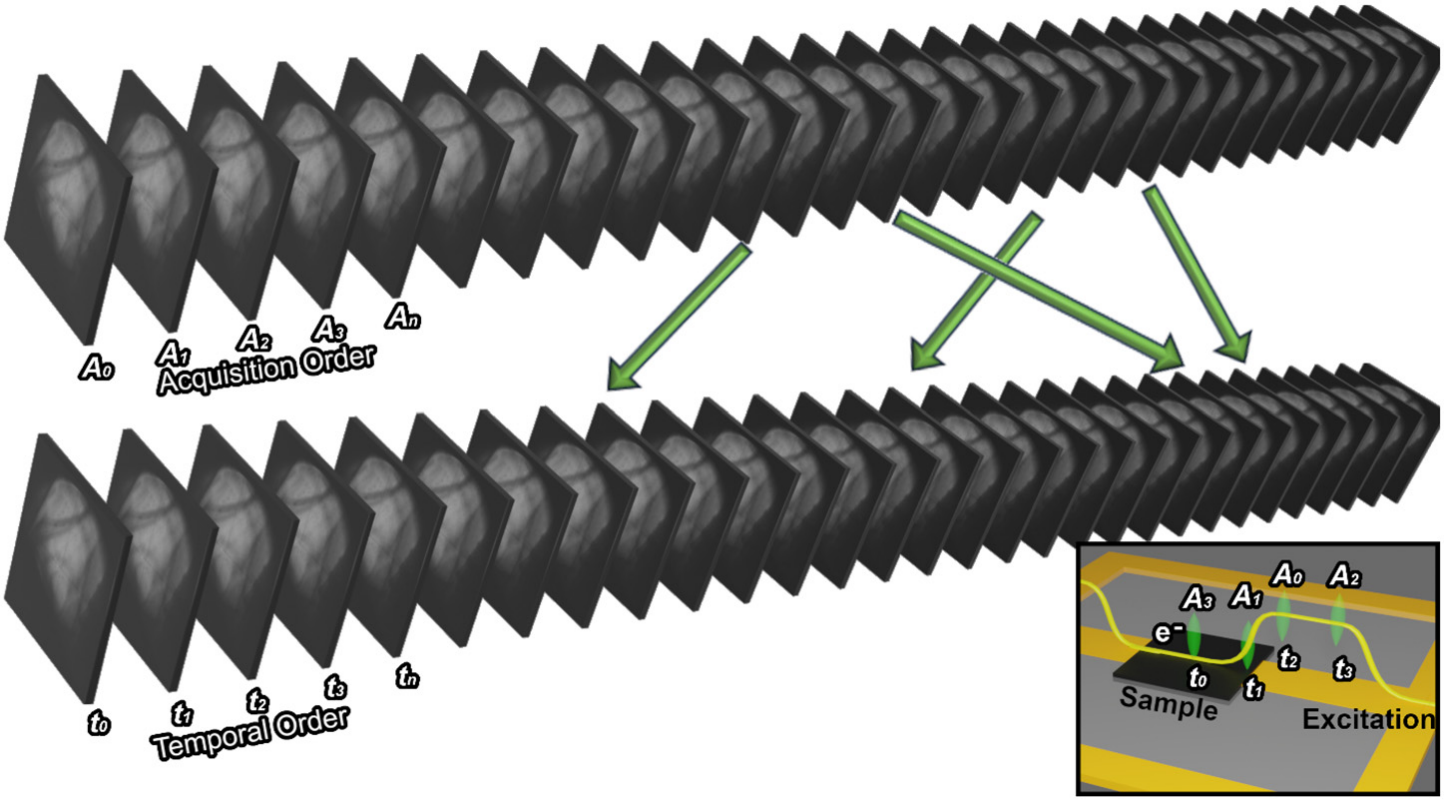
Demonstration of image acquisition in a randomized order by changing the delay mechanism so the collection does not have temporal ordering. For instance, in a four-image acquisition (inset), the earliest time-point is *t_0_* and latest is *t_4_*, but here they are the fourth and third acquisitions, respectively. The images are reordered during post-processing to the correct temporal order to remove long-term artifacts, which may dominate the image sequence. In the inset, the yellow square wave represents a pulsed excitation on the sample, and green represents the time-dependent position of the electron beam for each image acquired.

Turning to the elucidation of time-resolved dynamics, we will first present a technique that is ubiquitous among other techniques in materials science but has been undervalued in UEM publications. In UEM, each image is a static snapshot of a single point in the time-resolved response of the material, due to the stroboscopic nature of the technique, making the order of the acquisitions irrelevant. We have a custom software to randomly shuffle the order of each time-point acquisition over the temporal region of interest (i.e., 0–200 ns). We recognize that the innovation in this work is not in the randomization scheme or the process, but instead the data processing that is enabled by this procedure. Figure 3 is an illustration describing the randomization process containing two sequence orders: the acquisition order and temporal order. The acquisition order is dictated by the sequence that each image is acquired, such that *A_0_*, *A_1_*, and *A_2_* are the first, second, and third image acquired, respectively. The temporal order indicates the delay that each image corresponds to, irrespective of acquisition order. The example in the inset of Fig. 3 shows that the time point, *t_0_*, corresponds to when the excitation arrives at the sample, even though this image was collected fourth in the dataset. The unscrambling process occurs during post-processing by reordering the data in the temporal order for data visualization purpose.

**FIG. 4.**
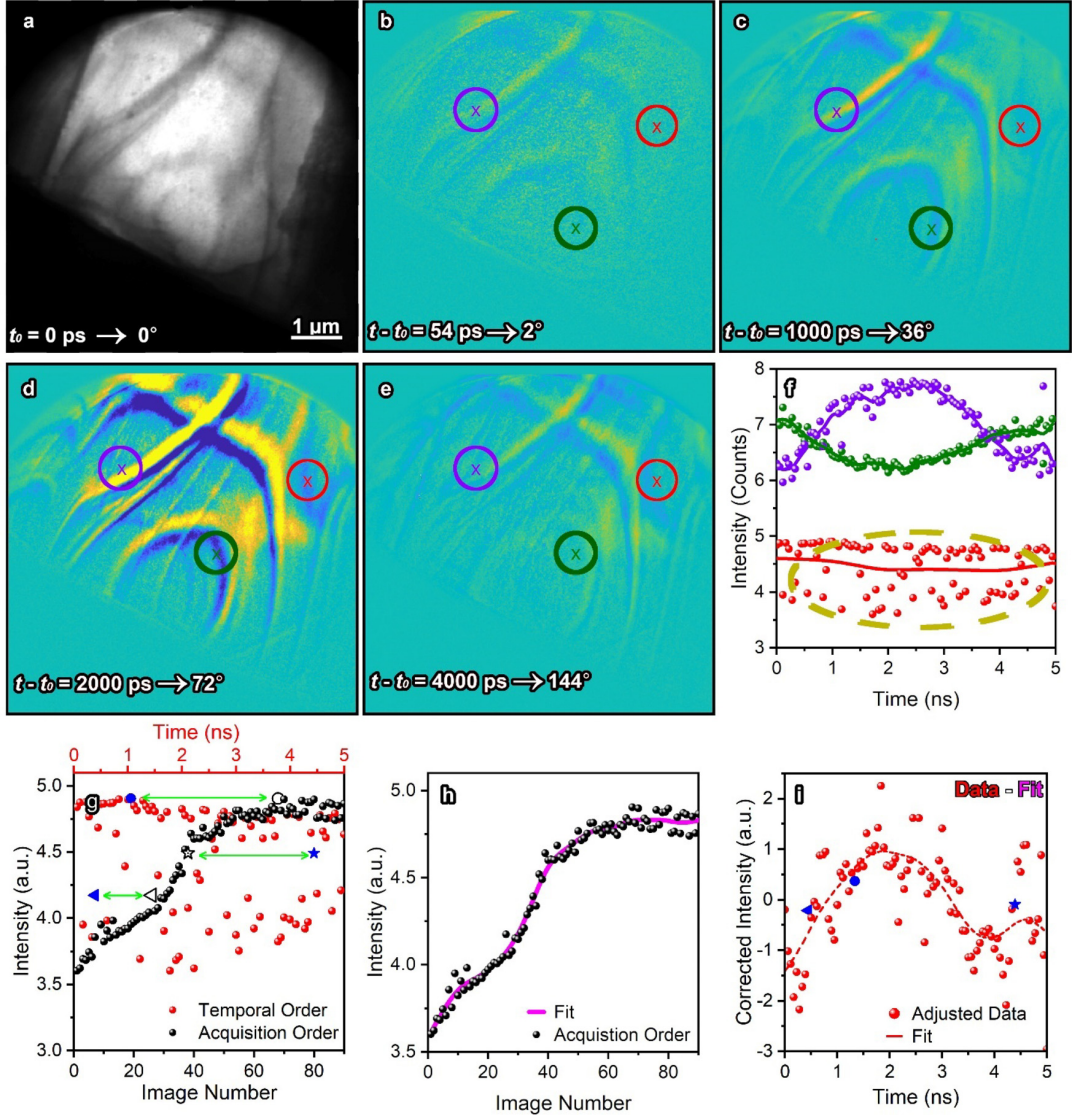
Extraction of time-resolved dynamics from low-signal datasets using a randomized image sequence. (a) Bright field reference image of a LiNbO_3_ lamella at the start of one cycle of a ±2.5 V, 200 MHz excitation. (b)–(e) Difference images subtracting the designated time-points from the reference image. (f) Single-pixel, unprocessed data extracted from the color coded locations in the difference images. The yellow oval indicates data extracted from a pixel with high noise levels. (g) Extracted time-ordered data from the red position also shown in (f) with respect to the acquisition order overlayed in black. Three data points, represented in blue, have been selected to demonstrate the correction of the data throughout. Green arrows indicate the positional difference for each selected data point between the temporal and acquisition order sequences. (h) Spline fit applied to the acquisition order image sequence. (i) Corrected intensity of the data in (g) with three blue data points demonstrating the new intensity they correspond to after correction. The red dashed line is a moving mean of the corrected data, providing more insight to the underlying mechanisms. Note that in electrically driven UEM, data are collected from 0 to 2*π* for a 200 MHz frequency (5 ns), so here we are observing one wave front of the excited SAW.

Once the randomized image slices have been acquired and reorganized into the correct temporal sequence, interpretation and analysis is necessary to extract the resulting physics from the data. Here, we used a ±2.5 V, 200 MHz excitation, which launches a propagating SAW from the IDT toward the electron transparent region at the sample edge during the positive potential of each period. This results in a single wavefront for the entire 5 ns period as shown in Fig. 4. Supplementary material Video 2^62^ contains the time-ordered dynamics observed during the SAW, which is propagation through the sample after minimal preprocessing (see the supplementary material for preprocessing procedures including template matching and normalization). Throughout the 4 h experiment, the electron beam drifted by about 25 pixels. After the derandomization process is applied to the image stack, the electron beam appears chaotic and is known to be an artifact. However, this reveals the time-dependent responses of the bend contours as they interact with the incoming SAW. Figures 4(a)–4(e) contain the reference image at time-zero along with four difference images at interesting time-points during the image sequence. For clarity, the time-points displayed here were selected because they were neighbors during the acquisition order, but distant from one another in the temporal sequence. Notably, the difference images show that the sample distorts to accommodate the propagating SAW and then returns to the initial state. Additionally, three types of dynamic processes can be extracted from the time-resolved dynamics from different levels of interpretation difficulty, represented by the purple (high signal and low noise), green (low signal and low noise), and red (low signal and high noise). Here, we focus on the low signal, high noise contrast features because we have developed an algorithm specifically designed to extract useful information from these types of datasets. Figures 2(g)–2(i) contain an extremely noisy dataset (red) and display the position of three selected data points in relation to the temporal and acquisition orders. We then apply a rough spline fit to the acquisition order image sequence [Fig. 2(h)], which creates a background signal that corrects the data to the final results [Fig. 2(i)].

Out of the three single-pixel intensity measurements here, two responses contain more distinguished increases and decreases over time. This indicates that the local structure factor aligns more strongly or weakly to the initial Bragg condition, presumably because the SAW modulates the local tilt of the sample relative to the electron beam. In this experiment, we intentionally selected datasets with large fluctuations in the temporal order sequencing for visualization purpose and to demonstrate that our method succeeded in pulling valuable signals from data that would ordinarily be too noisy for analysis. The physical mechanism for the contrast variation is reserved for another publication because the goal of this discussion is to focus on the algorithm that extracts low-signal phenomena. Without the randomized process utilized here, the intensity rise in Fig. 4(h) would overwhelm the dynamics. One thing to note is that this randomization process can shuffle the images such that the temporal and acquisition order have minimal difference. If this occurs, the background removal in Fig. 4(h) also would contain the acoustic phonon dynamics. In the supplementary material,^62^ we have calculated the probability of this happening to be approximately 7.3 × 10^−4^ for this experiment. Additionally, the probability of the failure approaches zero as the number of images in the dataset increases. Therefore, by randomizing the acquisitions and applying our fitting methods, we can process contrast modulations suffering from low signal to noise and be confident that the processing is not distorting the data. These extraction procedures reveal a single period of the traveling SAW, which is at the position indicated in red.

**FIG. 5.**
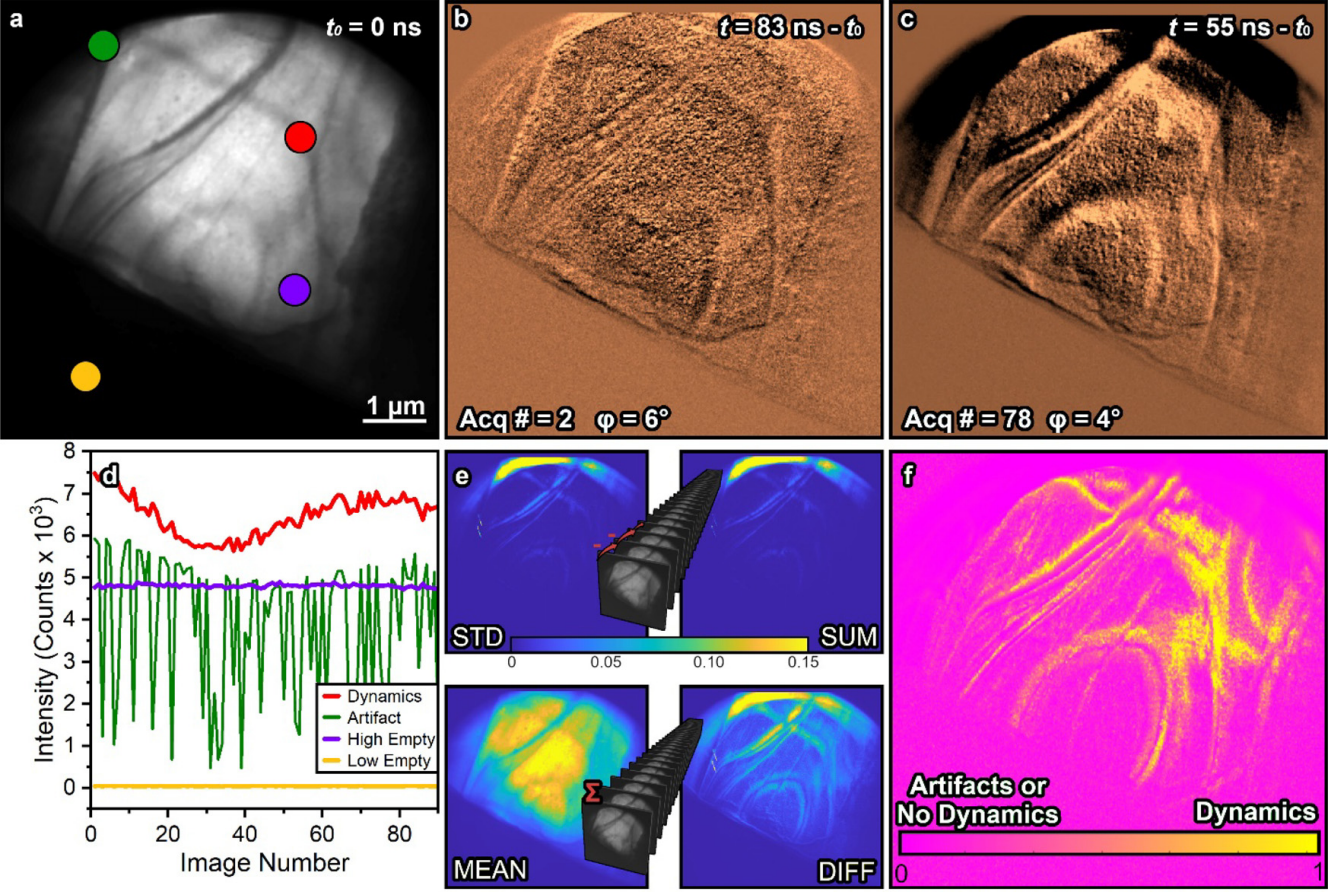
Methodology for extracting useful time-resolved dynamics from chaotic and/or noisy data sets. (a) Bright field image using the reference image, *t*_0_ = 0 ns. (b) and (c) Difference images of the indicated time point after subtracting the reference image with the acquisition order number and phase indicated. (d) Time-varying intensity profiles taken from the color-coded positions in (a). Specifically, there are four types of responses in a UEM dataset, including combinations when the artifacts and dynamics interfere, such as Fig. 4. (e) Examples of different statistical images for the local (top) and global (bottom) time-varying responses of the sample. (f) Constructed metric used to distinguish artifacts from dynamics using the statistical images in (e). Note that a thorough description of the methods is described in the supplementary material.^62^

Once we are able to extract low-signal dynamic responses from complicated data structures with varying levels of noise, we opted to explore the ability to separate time-resolved responses of the sample from intermingled artifacts. Figure 5 contains the algorithm (see the supplementary material for more details) used to create a “dynamic map” of the LiNbO_3_ response. Figures 5(b) and 5(c) contain two difference images that are adjacent to each other in the temporal order but separated by 76 images (over 1 h) during the acquisition. The notable difference is the broad black region at the top of Fig. 5(c) present because the electron beam drifted throughout the experiment. When the beam drifts into a region without the initial intensity, the change overwhelms the data processing. However, we have distinguished four types of intensity responses in every UEM dataset, which we use to our advantage to validate the time-resolved dynamics that are trustworthy. The ROIs in Fig. 5(a) are correlated with extracted signals containing artifacts (green), dynamics (red), and unchanging results (purple, yellow) of varying intensity. As a result, we developed a novel metric that evaluates the time-varying intensity of each independent pixel for the probability that it is containing useable time-resolved responses, as shown in Fig. 5(f). The resulting metric is composed of creating statistical images of the global (extrema of entire dataset) and local (effect of neighboring images on one another) differences of the time-ordered dataset after derandomization. Specifically, we constructed numerous statistical images, including the sum, standard deviation, and average, which were used to construct the pixel-by-pixel dynamic map. An in-depth calculation and description for the calculation of Fig. 5(f) are available in the supplementary material.^62^

After statistical evaluation of every pixel in the resulting UEM image sequence, we have been able to develop a new metric that validates real time-resolved responses of the sample and separates them from artifacts. Specifically, it enables the researcher to disregard unreliable results, which may have been inadvertently acquired due to the longevity of UEM experiments. Additionally, the developed algorithm only relies on statistics contained within the image sequence with minimal processing, leading to a significant reduction in biases when evaluating the map of the time-resolved dynamics. Here, we observe that the algorithm indicates that the bend contour motion contains the most reliable responses to the traveling SAW. However, the algorithm also rejects drifting motion of the electron beam because it is a consequence of the instrument stability and not attributed to the SAW, which could be improperly assigned by a less familiar researcher. Instead, in the supplementary material, we have provided additional testing of this procedure on our previously published UEM datasets and comment on the locations that the analysis could have been improved if this metric had been developed previously.

**FIG. 6.**
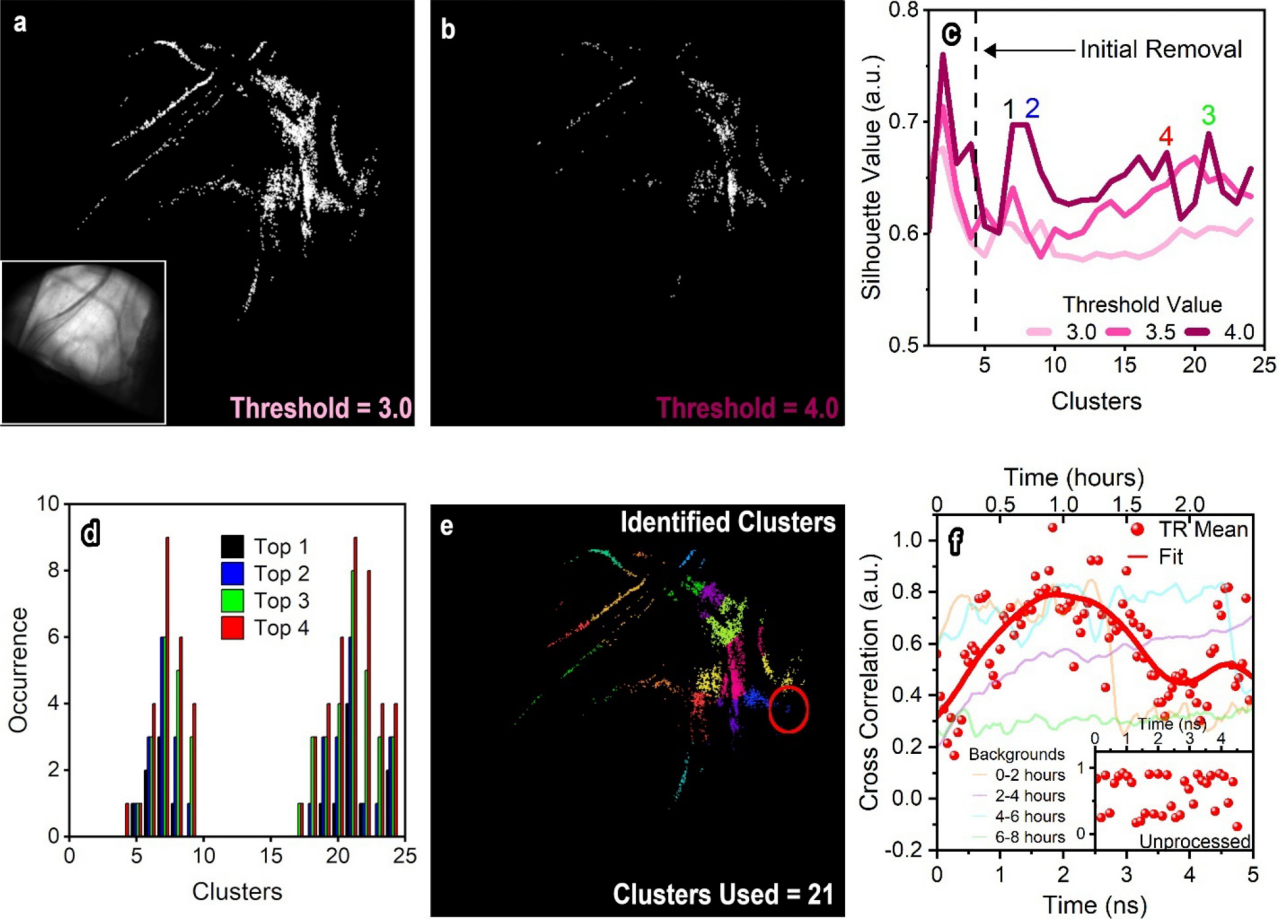
Example methodology to reduce researcher bias when interpreting complicated datasets with low signal to noise. Binarized images of the dynamic response metric in Fig. 5(f) using threshold values of 3.0 (a) and 4.0 (b). The inset is the reference image at *t* = 0 ns. (c) Calculation of silhouettes for binarized images with different thresholds. Note that in application, 25–50 silhouette measurements are constructed, but only three are displayed for simplicity. The highest four amplitude peaks for the 4.0 threshold image have been indicated. (d) Histogram of the highest intensity peaks found from all silhouette measurements as a function of total number of clusters. The peaks at 7 and 21 clusters indicate that the best segmentation of the UEM dynamics fit into these quantities of clusters. (e) Binarized image with the data segmented into 21 different spatially selective clusters. We selected to evaluate the data using 21 clusters because it is more reliable to manually combine clusters than attempt to break them apart for obvious anomalies that the algorithm cannot catch, even though this is a subjective decision. (f) Final extraction of low-signal time-resolved response when a SAW travels through the LiNbO_3_ sample. The inset provides the unprocessed data for comparison. The faint curves in the background are background intensity profiles that are removed due to the randomization process. Some of these clear changes would be very easy to misconstrue as time-resolved dynamics.

To finish this publication, we provide one example of further analysis that is enabled because of our dynamic map algorithm. All UEM analyses cover a side range of phenomena data types, artifacts, and dynamics, so the following is not intended to be considered a best practice or a flawless method but is an add-on to the dynamic map algorithm, which should be the primary focus. Figure 5 is a minimally expensive example that demonstrates the use of the algorithm to alleviate many of the initial processing techniques, which compromise the analysis with researcher biases. We hope this segment motivates dedicated artificially intelligent models for each UEM experiment because different materials systems require different analysis methods and complexity. In many instances, researcher bias manifests itself in UEM experiments in the form of selecting specific regions of the data that support the expected story or adjusting the analysis to reduce noise and amplify signal. In many instances, the interpretation of UEM data could support far greater physical insights with the assistance of statistics and automated computing. For example, in Fig. 4, we selected three single-pixel locations to evaluate, each providing a drastically different interpretation of the results. In fact, the results in Fig. 4 were repeated multiple times, and the highest quality measurements were used for this publication. In most UEM datasets, there are significant results buried beneath the face-value interpretation. Figure 6 contains one example capable of reducing researcher bias on the analyses for UEM datasets. Fortunately, we used k-clustering via the silhouette evaluation method (full details in the supplementary material^62^), which is well established and simple to implement for the purpose of this publication, while other experiments will require custom analysis.^59–61^ Here, we binarized the metric developed in Fig. 5(f) using different thresholding values from 0.5 to 5 (unfortunately, this thresholding range still requires a researcher bias). Then, the silhouette values were calculated for numerous binarized images for *k*-clustering algorithms ranging up to 25 clusters (maximum being another bias). Additionally, the silhouette method for k-clustering regularly yields high silhouette values for low cluster quantities. Due to this, we have ignored the first four clusters so that the distributions in Fig. 6(d) are not centered around 1 cluster, ultimately inducing another significant researcher bias. For each binary image, the top four peaks of each silhouette were numerically sorted by decreasing magnitude and placed the results into the corresponding histogram [Fig. 6(d)]. Note that when only the largest peak is observed, it does not have a significantly different structures to 2, 3 or 4 peaks, so additional data compilation strengthens the interpretation of the data. Afterward, we utilized a basic *k*-clustering algorithm to separate the 21 clusters that were statistically identified as the most prominent sources of time-resolved dynamics and used the result to extract the resulting low-signal response shown in Fig. 6(f). Note that we ignored the peak corresponding to the lower quantity of clusters, which is also another researcher bias.

Finally, we have provided an example analysis that attempts to reduce researcher bias in the interpretation of UEM datasets. There are still flaws in the procedure, which should continue to be addressed and adjusted to pertain to other experiments. First, even through rigorous statistical attempts to extract the results, human judgment is required when deciding which cluster is considered the most important, or how each cluster interferes or is coupled to others. Therefore, artificial intelligence and machine learning are expected to play a major role in the advancement of UEM analysis. For now, these methods are not suitable for non-imaging techniques such as diffraction because the signal is concentrated to specific locations. Specifically, drift of the selected area aperture or sample during UEM experiments can cause extreme consequences when evaluating the data causing the dynamic map algorithm to fail. However, we believe our dynamic map is a substantial step forward for UEM because many systematic controls and verification processes are undervalued in the field.

## IV. SUMMARY AND CONCLUSIONS

Overall, we have observed an electrically excited SAW traveling from the edge of an IDT structure and passing through an electron transparent region of the sample, which modulates the local structure when interacting with the wavefront. We have also provided a novel metric that constructs a dynamic map capable of providing a statistical analysis of the time-resolved dynamics present in any imaging based UEM dataset. This new metric allows us to analyze and evaluate data that would otherwise be too noisy to be useful. We also demonstrated the effectiveness of the dynamic map by using it in conjugation with a classical k-clustering technique that provides a reduction of researcher bias during data analysis. We hope that these procedures, analyses, and results will provide substantial assistance in interpreting and evaluating complicated UEM datasets, specifically because of the enormous amounts of time and care that must accompany each experiment.

## SUPPLEMENTARY MATERIAL

See the supplementary material for a more thorough description of the algorithm to distinguish between anamalous results and time-resolved dynamics in the ultrafast electron microscopy data sets. Additionally, there are descriptions pertaining to image preprocessing, validation of the randomization process and a demonstration of a complete data set. The algorithm has also been tested on two other data sets we previously published and identified additional dynamic responses that were previously unknown.

## Data Availability

The data that support the findings of this study are available from the corresponding author upon reasonable request.
